# Comparative Transcriptomics Unravels Prodigiosin's Potential Cancer-Specific Activity Between Human Small Airway Epithelial Cells and Lung Adenocarcinoma Cells

**DOI:** 10.3389/fonc.2018.00573

**Published:** 2018-12-05

**Authors:** Bala Davient, Jessica Pei Zhen Ng, Qiang Xiao, Liang Li, Liang Yang

**Affiliations:** ^1^Singapore Centre for Environmental Life Sciences Engineering, Nanyang Technological University, Singapore, Singapore; ^2^School of Biological Sciences, Nanyang Technological University, Singapore, Singapore; ^3^Respiratory Medicine, Shunde Hospital, Southern Medical University, The First People's Hospital of Shunde Foshan, Foshan, China; ^4^Shenzhen Institute of Advance Technology, Chinese Academy of Sciences, Shenzhen, China; ^5^School of Medicine, Southern University of Science and Technology, Shenzhen, China

**Keywords:** prodigiosin, small molecule, chemotherapy, lung cancer, selective, RNA-sequencing

## Abstract

**Objective:** Non-Small Cell Lung Cancer (NSCLC) is extremely lethal upon metastasis and requires safe and effective systemic therapies to improve a patient's prognosis. Prodigiosin (PG) appears to selectively and effectively target cancer but not healthy cells. However, PG's cancer-specific activity has remained elusive until recently.

**Methods:** PG's cancer-specific performance was compared to Docetaxel (DTX), Paclitaxel (PTX), and Doxorubicin (DOX) against human lung adenocarcinoma (A549) and human small airway epithelial cells (HSAEC). Combination of PG with DTX, PTX, or DOX in a 1:1 ED50 ratio was also evaluated. MTT assay was used to determine the post-treatment cell viability. RNA-sequencing was used for comparative transcriptomics analysis between A549 and HSAEC treated with 1.0 μM PG for 24 h.

**Results:** PG reduced A549 cell viability by four-folds greater than HSAEC. In comparison to DTX, PTX and DOX, PG was ~1.7 times more toxic toward A549, and 2.5 times more protective toward HSAEC. Combination of PG in a 1:1 ED50 ratio with DTX, PTX, or DOX failed to exhibit synergistic toxicity toward A549 or protection toward HSAEC. In A549, genes associated in DNA replication were downregulated, while genes directly or indirectly associated in lipid and cholesterol biogenesis were upregulated. In HSAEC, co-upregulation of oncogenic and tumor-suppressive genes was observed.

**Conclusion:** An overactive lipid and cholesterol biogenesis could have caused A549's autophagy, while a balancing-act between genes of oncogenic and tumor-suppressive nature could have conferred HSAEC heightened survival. Overall, PG appears to be a smart chemotherapeutic agent that may be both safe and effective for NSCLC patients.

## Introduction

Cancer represents a major disease burden to mankind (1–4), and it accounts for almost one out of six deaths worldwide (5). Out of the 8.8 million cancer deaths in 2015, 1.69 million was due to lung cancer (5). The high mortality in patients with lung cancer is often associated with an advanced metastatic disease state (6, 7). In such cases, effective systemic therapies are vital to improve a patient's prognosis. Targeted therapy, immunotherapy and chemotherapy are all systemic therapies, each with their own strengths and weaknesses.

Targeted therapies can mitigate most side-effects commonly seen in chemotherapy by working on specific mutations unique to cancer cells (8), but their highly specific nature excludes patients whom do not harbor these mutations (9). Almost 80% of all lung cancers are Non-Small Cell Lung Cancer (NSCLC). The most studied target for NSCLC is the Epidermal Growth Factor Receptor (EGFR). There exist three classes of activating EGFR mutations that sensitizes NSCLCs to EGFR Tyrosine Kinase Inhibitors (TKIs). These activating EGFR mutations have been well summarized in the literature (10). Gefitinib, Erlotinib, Afatinib, Osimertinib, and Dacomitinib are a few prominent and promising EGFR TKIs used in NSCLC patients harboring specific activating EGFR mutations. Gefitinib and Erlotinib are inhibitors of a few specific EGFR mutations found in some NSCLC patients and have demonstrated enduring progression free survival for responders (11–13). Although effective, Gefitinib, Erlotinib, and the other EGFR TKIs are beneficial to only a small population of patients as only about 15% of Caucasian and 50% of Asian lung adenocarcinoma patients harbor EGFR mutations (14, 15).

Immunotherapy exploits the patient's own immune system against cancers (16), but its success depends on the cancer's ability to display its unique neoantigens on its outer cell membrane (17–19) to be identified and destroyed by immune cells (20). Cancers can evade immune destruction by expressing Programmed Death (PD) Ligand 1 (PD-L1), which binds to PD-1 receptors on CD8+ T-cells, inhibiting cytotoxic elimination (21). Nivolumab and Pembrolizumab are antibodies against PD-1. Their prevention of interaction with PD-1 allows CD8+ T-cells to eliminate cancer cells such as NSCLCs (22, 23). Anti-PD-1 effectiveness against NSCLC has been reported to positively correlate with the cancer cell's mutation burden, as a high mutation load generates unique neoantigens for T-cell recognition (24). However, response rates of anti-PD-1 in NSCLC patients appears to be low at ~19% (22, 23, 25).

In contrast to targeted and immunotherapy, chemotherapy offers broader patient coverage and is still the mainstream cancer therapy available for the majority of cancer patients (26). Platinum-based doublet chemotherapies have been indicated as the first-line against NSCLC with response rates ranging from 25 to 35% (27, 28). However, despite better response rates, their inability to distinguish rapidly diving cancer cells from healthy cells could lead to debilitating side-effects such as anemia, nausea, and neurotoxicity (29).

NSCLC urgently require therapies that are effective, have wide coverage, and harbor fewer side effects. Many studies are ongoing to improve systemic therapies for metastatic NSCLC. In terms of chemotherapies, the search for newer and safer treatments, alone or in combination, persists (30–33).

Nature provides a rich source of anti-cancer agents suitable for chemotherapy. Docetaxel (DTX), Paclitaxel (PTX), and Doxorubicin (DOX) are natural compounds that have been used against NSCLC (34, 35). Recently, Prodigiosin (PG), a secondary metabolite from *Serratia marcescens*, was observed to inhibit NSCLC proliferation (36). Interestingly, PG has been reported to exhibit high cancer-specificity (37–39). This means that PG could potentially mitigate common side-effects associated with chemotherapies, making it a smart chemotherapy candidate.

The current understanding of PG's anti-cancer mechanisms of action encompasses cytoplasmic acidification through modulation of H^+^/Cl^−^ symporters, DNA damage through copper-mediated oxidative cleavage, inhibition of topoisomerases, and ATP synthesis reduction through disruption of the mitochondrial proton gradient (40). At the molecular level, PG has been described to initiate autophagy through mTOR deactivation (39) and apoptosis through the disruption of BCL-2 family pro-survival members (39, 41) or downregulation of pro-survival Survivin (40, 42), a member of the inhibitor of apoptosis. In addition, common to many cancers is the dysregulation of p53, a protein that dictates cell survival or cell death upon cell stress. In most cancers, p53 activity is lost and cells attain a permanent survival status. In some reports, PG was able to induce cancer cell apoptosis in a p53-independent manner (43, 44). This reveals that PG could trigger alternative apoptosis pathways.

Altogether, PG appears to be a promising chemotherapeutic agent which warrants further research into its mechanisms of action. At present, there exists limited data on PG's mechanisms of action to draw meaningful links between studies. Here, we add value to the current knowledge by unveiling PG's potential cancer-specific activity through comparative transcriptomics analysis between Human Lung Adenocarcinoma (A549) and Human Small Airway Epithelial Cells (HSAEC), with Human Colorectal Carcinoma Cells (HCT116) as a cancer control. In addition, we also report on PG's *in vitro* effectiveness and safety, based on the degree of cancer cytotoxicity and selectivity, respectively, in comparison to DTX, PTX and DOX.

## Materials and Methods

### Materials

Docetaxel purum (DTX), doxorubicin hydrochloride (DOX), paclitaxel from *Taxus brevifolia* (PTX), prodigiosin hydrochloride from *Serratia marcescens* (PG), and dimethyl sulfoxide (DMSO) were purchased from Sigma (St. Louis, MO, USA). 3-(4,5-Dimethylthiazol-2-yl)-2,5-diphenyltetrazolium bromide (MTT) was purchased from Bio Basic (Amherst, NY, USA). Proteinase K, RNase-Free DNase I and the RNAprotect Cell Reagent were purchased from Qiagen (Hilden, Germany). TURBO™ DNase, Qubit™ dsDNA HS, and RNA HS Assay Kits were purchased from Invitrogen (Waltham, MA, USA). Angencourt RNAClean XP Kit was purchased from Beckman Coulter (Bera, CA, USA). RNA ScreenTape was purchased from Agilent (Santa Clara, CA, USA).

### Cell Culture

Primary Small Airway Epithelial Cells; Normal, Human (HSAEC) (ATCC® PCS301-010™), A549 (ATCC® CCL-185™), HCT116 (ATCC® CCL-247™), and the Airway Epithelial Cell Basal Medium (AECBM) with associated growth factors were purchased from the American Type Culture Collection (ATCC) (Manassas, VA, USA). Phosphate Buffered Saline (PBS) without calcium and magnesium, high glucose Dulbecco's Modified Eagles Media (DMEM) with added L-glutamine, sodium pyruvate, and phenol red, were purchased from GE Healthcare Life Sciences (Logan, UT, USA). Heat-inactivated Fetal Bovine Serum (FBS) of South American origin and Trypsin-EDTA (0.25%) with phenol red were purchased from Gibco (Waltham, MA, USA). HSAEC cells were cultured with 8 mL AECBM while both A549 and HCT116 cells were cultured with 8 mL DMEM supplemented with 10% FBS, which henceforth will be referred to as complete media, in a 75 cm^2^ culture flask. All culture flasks were incubated in a humidified atmosphere at 37°C with 5% CO_2_. All incubations mentioned henceforth will be referring to these conditions. No *Mycoplasma* testing was performed.

### Cell Viability Assay

DTX, PTX, DOX, and PG were reconstituted with DMSO to a stock concentration of 50, 50, 80, and 2 mM, respectively. Drugs were diluted in pre-warmed AECBM or complete media of 37°C. For each drug concentration tested, an equivalent DMSO concentration was created as control (Supplementary Figure S1).

At ~90% cell confluency, cells were split into 96-well flat-bottomed plates at a seed density and final volume of 7,000 cells and 100 μL per well. Cultures were incubated overnight for 24 h. At ~80% confluency, the spent media was replaced with either the treatment or control media to a final volume of 100 μL per well. The culture plates were incubated for another 48 h.

The MTT shipped in the powdered state was reconstituted with PBS to a final concentration of 5 mg/mL and sterile filtered with a 0.2 μm Acrodisk Syringe Filter (PALL, Port Washington, NY, USA). This was mixed at a 1:1 ratio with serum-free DMEM or AECBM to create the MTT mix. After the 48 h of treatment, the spent drug media was replaced with 100 μL of the MTT mix. The cultures were incubated for an additional 3 h before being homogenized with 150 μL of DMSO. Cell viability was measured with the Infinite® M200 Pro (Tecan, Männedorf, Zürich, Switzerland) microplate reader at 590 nm.

### Drug Cytotoxicity Screening

HSAEC and A549 cells, both at passage P6, were split into three 25 cm^2^ culture flasks. These cultures were propagated further for two more passages, and at P8, each cell line was considered to have three biological replicates of *n* = 3 (45). The cells were thereafter cultured in 96-well plates as technical duplicates per biological replicate.

DTX, PTX, DOX, and PG's ED50 were pre-determined with A549 cells (Supplementary Figure S2). The ED50 for DTX, PTX, DOX, and PG were 0.1, 0.1, 1, and 0.3 μM, respectively. For the combination therapies with PG, drugs were mixed in a 1:1 ED50 ratio. All treatments were first created as eight-fold stock concentrations and were serially diluted by two-folds (i.e., 8:8 to 4:4 till 0.25:0.25). All other steps conducted have been described under the “Cell Viability Assay” section.

### RNA Extraction and Quality Controls

HSAEC, A549, and HCT116 at passage number P8 were cultured as technical triplicates in 25 cm^2^ culture flasks, and after two more passages, each cell line was considered to have biological triplicates of *n* = 3 (45). At 90% confluency, HSAEC and A549 cells were split at a seed density of 3.0 × 10^4^ cells/cm,^2^ while HCT116 cells were split at 6.0 × 10^4^ cells/cm^2^ into 6-well plates. After 24 h of incubation in 3 mL of AECBM or complete media, the spent media was replaced with 3 mL of either 1.0 μM PG (treatment) or 0.05% DMSO (control). Cells were incubated for another 24 h and thereafter, the media was replaced with 1 mL of RNAprotect Cell Reagent.

Cells were gently agitated on an orbital shaker at 80 revolutions per minute for 10 min. A lysis cocktail comprised of 10 μL 1% β-mercaptoethanol, 20 μL proteinase K, and 800 μL RLT buffer, which was a component from the RNeasy Mini Kit (Qiagen), was homogenized with cells in each well. The RNA extraction was conducted according to instructions found in the RNeasy Mini Kit.

A 30 min on-column DNase I treatment was performed. DNA contamination was further minimized with TURBO™ DNase treatment. Once RNA was purified with the Angencourt RNAClean XP Kit, RNA integrity was verified using the RNA ScreenTape with analysis on the Agilent 2200 TapeStation (Agilent). Using the Qubit™ dsDNA HS and RNA HS Assay Kits, total RNA was quantified fluorometrically via the Qubit™ Fluorometer 2.0 (Invitrogen).

### RNA Sequencing and Data Processing

RNA library preparation and sequencing were conducted by an in-house facility at Singapore Centre for Environmental Life Science Engineering (SCELSE). Briefly, library preparation was executed with the Illumina® TruSeq® Stranded messenger RNA Sample Prep Kit (Illumina, San Diego, CA, USA). The output which was cDNA fragments were paired-end sequenced at read lengths of 100 nucleotides via the Illumina® HiSeq 2500 (Illumina) platform.

All samples had a sequencing depth of more than 24 million reads. These reads were processed using the CLC Genomics Workbench Version 11.0.1 (CLC Bio, Aarhus, Denmark). The default settings were used unless otherwise stated. All reads were trimmed with a quality score of 0.05. Using the “RNA-Seq Analysis” function, the trimmed reads were mapped onto the human genome GRCh38 downloaded from the Ensemble database. The maximum number of hits for a read was set to 1. Gene hits were annotated with GRCh38.92 acquired from the Ensemble database. Gene expression was measured as total counts, where each paired-read was considered as 1. A negative binomial test was performed using the workbench's “Differential Expression for RNA-Seq” tool to establish the differentially expressed genes (DEGs). All raw and processed sequence files may be acquired from Gene Expression Omnibus (Accession number: GSE118448).

### Functional Analysis

DEG datasets were exported from CLC into the Ingenuity® Pathway Analysis (IPA; Qiagen) Version 44691306 software. A Log2 Fold-change (Log2FC) of ±1 with a false discovery rate (FDR) adjusted *p*-value of < 0.05 was applied to the datasets. With these cut-off values, HSAEC had 2,222, A549 had 2,004, and HCT116 had 2,199 DEGs out of 37,258 successfully annotated gene identifiers.

### Statistical Analysis

The Welch two-tailed *t*-test available in GraphPad Prism 8 was applied onto the drug cytotoxicity screening assay datasets. This statistical test considers the data to have been sampled from a Gaussian population but does not presume that the two populations under scrutiny have the same standard deviation. The null hypothesis is defined as the two populations tested having equal means. When *p* > 0.05, the null hypothesis is not rejected, and the interpretation would be that the evidence is not convincing enough to claim that the means of the two populations tested are different.

## Results

### PG Demonstrated Selective Toxicity Toward A549 but not HSAEC

**Figure 1:**
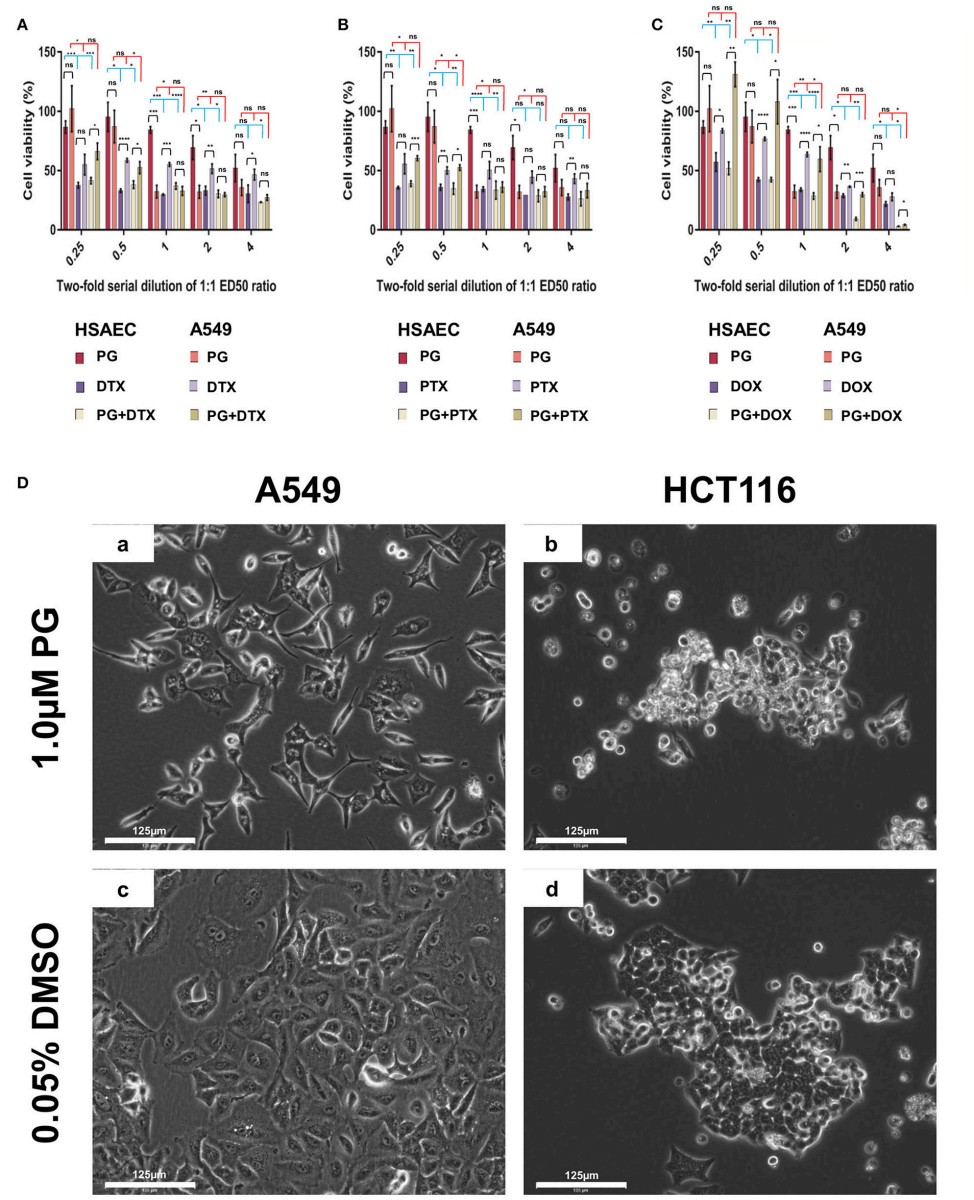
Cell viability of HSAEC and A549 cell measured by the MTT assay after 48-h of PG treatment **(A–C)**. Effects of 1.0 μM PG on A549 and HCT116 cell morphology after 24 h treatment **(Da–Dd)**. **(A)** PG, DTX, and PG+DTX. **(B)** PG, PTX, and PG+PTX. **(C)** PG, DOX, and PG+DOX. Bar graphs represent mean cell viability from biological triplicates (*n* = 3) while the black vertical lines on the bar tops represent standard deviation (SD). A Welch *t*-test was applied to the datasets; black horizontal lines compare drug effects between HSAEC and A549, blue lines compare within HSAEC, and red lines compare within A549 (*p < 0.05, **p < 0.01, ***p < 0.001, ****p < 0.0001, and “ns” is not significant). **(Da)** A549 and **(Db)** HCT116 were treated with 1.0 μM PG. **(Dc)** A549 and **(Dd)** HCT116 were treated with 0.05% DMSO as a negative control. Phase-contrast images were acquired at 20X magnification with the EVOS FL Auto 2 microscope. Images have not been enhanced. Scale bars represent 125 μm.

PG has been known to induce cancer cell death while preserving healthy cell's viability (37–39). Here, we evaluated PG's cancer-specific toxicity with cancer cell line A549 and immortalized human lung small airway epithelial cells (HSAEC; Figure 1). At PG's ED50 of 0.3 μM, cell viability of A549 was reduced by 67.7 ± 5.3%, while HSAEC was reduced by 15.6 ± 2.8%. As A549 is a cancer cell line while HSAEC is an immortalized healthy cell line, with both dividing rapidly, the greater reduction in A549 cell viability demonstrates PG's selective toxicity. PG concentrations >0.3 μM exhibited neither enhanced cancer toxicity nor healthy cell protection.

### PG Outperformed DTX, PTX, and DOX in Terms of Cancer-Specificity

Here, we define performance as the agent's ability to protect normal cells while being toxic to cancer cells. In other words, the degree of cancer-specificity. Evaluation of DTX, PTX, DOX, and PG's ED50 of 0.1, 0.1, 1.0, and 0.3 μM, respectively, against A549 and HSAEC, revealed PG's superior performance as a cancer-specific agent. At these concentrations, PG preserved HSAEC viability by 2.8, 2.4, and 2.5 times more than DTX, PTX, and DOX, respectively (Figure 1). Moreover, PG reduced A549 cell viability at an average of 1.7 times greater than the other agents.

### PG Exhibited Poor Performance in Combination With DTX, PTX, or DOX

DTX, PTX, or DOX in a 1:1 ED50 ratio with PG failed to exhibit anti-cancer synergism and were almost equally toxic, if not worst, toward HSAEC as compared to A549. 0.3 μM PG with 0.1 μM DTX reduced HSAEC viability by 63.0 ± 2.6% and A549 by 67.2 ± 3.7% (Figure 1A). 0.3 μM PG with 0.1 μM PTX reduced HSAEC viability by 66.4 ± 7.5% and A549 by 63.9 ± 4.3% (Figure 1B). 0.3 μM PG with 1.0 μM DOX reduced HSAEC viability by 71.4 ± 2.7% and A549 by 40.4 ± 10.4% (Figure 1C). PG in combination with DTX, PTX, or DOX, at 4:4, 2:2, 1:1, 0.5:0.5 or 0.25:0.25 ED50 ratio, failed to exhibit improved toxicity toward A549 with enhanced protection to HSAEC in comparison to 0.3 μM PG alone.

### PG Altered Both A549 and HCT116 Cancer Cells' Morphology

To determine if PG's anti-cancer activity can be observed beyond lung adenocarcinoma cells, in addition to A549 cells, we treated HCT116 cells, another cancer type which could serve as a cancer control, with 1.0 μM PG for 24 h prior microscopic visualization. A549 cells were found in low numbers, elongated, shriveled, with a deformed nucleus and non-homogenous cytoplasm (Figure 1Da). HCT116 cells appeared rounded-up, detached from culture surfaces, but still adhered to neighboring cells (Figure 1Db). Overall, PG demonstrated substantial morphological alterations in both A549 and HCT116 cancer cell lines.

### PG's Toxicity Possibly Mitigated Through a “Balancing Act” in HSAEC

**Figure 2:**
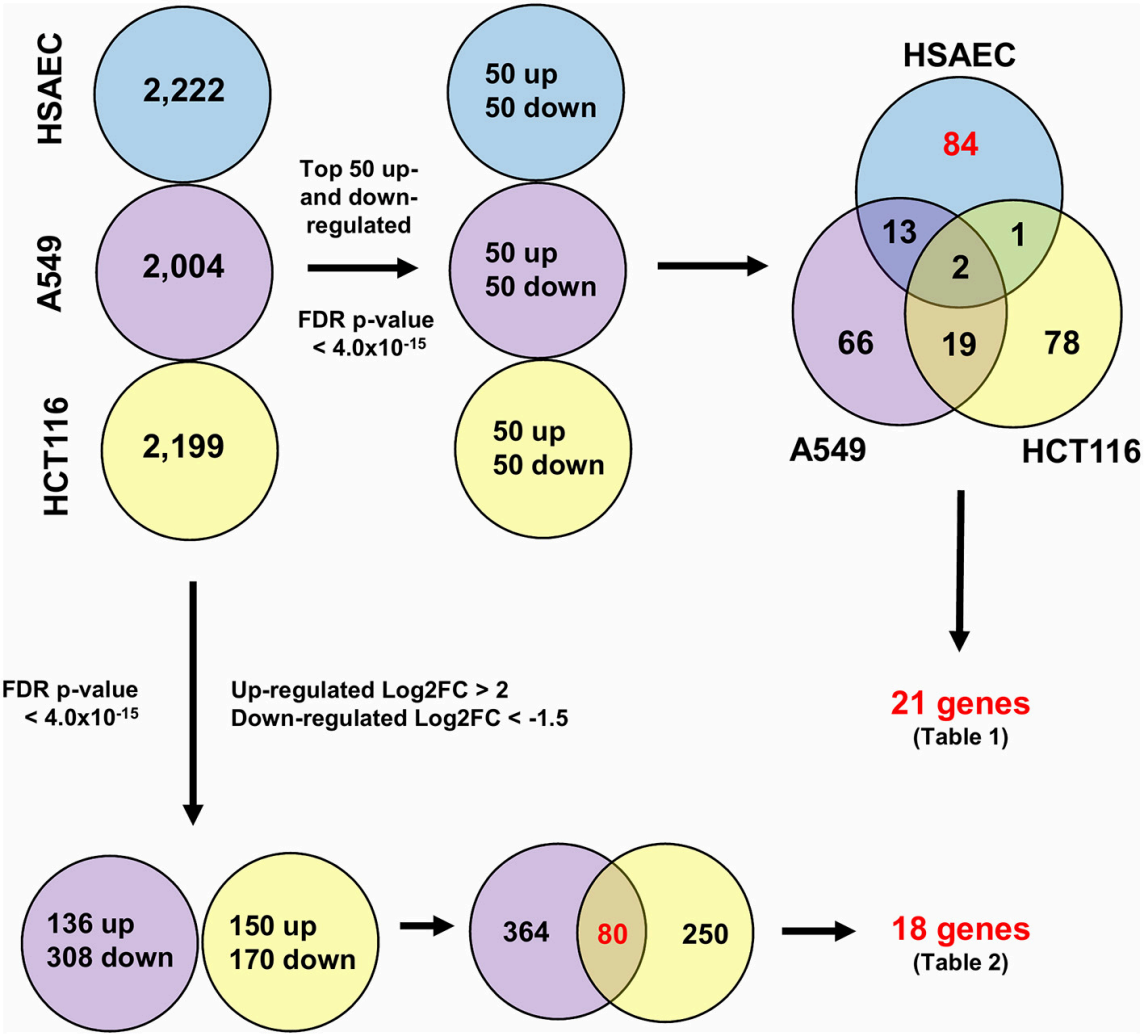
Differentially expressed genes from CLC Workbench for HSAEC, A549 and HCT116 after 24 h treatment with 1.0 μM PG. Blue circles represents HSAEC, purple as A549 and yellow as HCT116. Twenty one genes for Table 1 were derived from filtering 84 HSAEC-specific genes on the condition that corresponding genes in A549 and HCT116 had an FDR *p*-value < 0.05, and secondly, the difference of A549 and HCT116 gene expression in terms of Log2FC with HSAEC had to be > ± 1.5. Eighteen genes for Table 2 were derived from filtering 80 genes common to A549 and HCT116 on the condition that corresponding genes in A549 with HSAEC had an FDR *p*-value < 0.05, and secondly, the difference of A549 and HSAEC gene expression in terms of Log2FC with HSAEC had to be > ± 1.5.

To understand how PG protects healthy cells yet kills cancer cells, we conducted an RNA-sequencing experiment with HSAEC, A549 and HCT116 cells treated with 1.0 μM PG for 24 h. Using the top 50 up- and down-regulated genes per cell line, we were able to identify 84 DEGs specifically perturbed in HSAEC. These DEGs had an FDR *p*-value < 4.0 × 10^−15^ (Figure 2). For comparison validity, these 84 HSAEC-specific DEGs were filtered under two conditions. Firstly, the corresponding DEGs in A549 and HCT116 were required to have an FDR *p*-value < 0.05, and secondly, the difference in expression in terms of Log2FC with HSAEC had to be >± 1.5. Under these conditions, 21 DEGs were identified as fit for comparison (Table 1).

**Table 1:** HSAEC-specific DEGs in comparison with A549 and HCT116 cells after 24 h treatment with 1.0 μM PG.

			**Log2FC**		
**Gene name**	**Gene symbol**	**ENSEMBL ID**	**HSAEC**	**A549**	**HCT116**
**ONCOGENIC NATURED GENES**					
Pyruvate Dehydrogenase Kinase 4	PDK4	ENSG00000004799	6.87	1.25	1.62
Ras Related GTP Binding D	RRAGD	ENSG00000025039	4.92	2.03	0.68
Hes Related Family BHLH Transcription Factor with YRPW Motif 1	HEY1	ENSG00000164683	4.54	0.93	1.41
Tetraspanin 15	TSPAN15	ENSG00000099282	4.19	-0.61	1.28
Serpin Family B Member 9	SERPINB9	ENSG00000170542	3.69	-0.78	0.72
SHC Binding and Spindle Associated 1	SHCBP1	ENSG00000171241	-3.73	-2.18	-1.14
Carboxypeptidase A4	CPA4	ENSG00000128510	-3.68	0.49	0.67
Keratin 19	KRT19	ENSG00000171345	-3.48	1.97	0.76
Keratin 15	KRT15	ENSG00000171346	-3.28	1.13	2.00
Desmoglein 3	DSG3	ENSG00000134757	-2.86	–	–
**TUMOR-SUPPRESSIVE NATURED GENES**					
Metallothionein 1G	MT1G	ENSG00000125144	5.80	–	–
Metallothionein 1M	MT1M	ENSG00000205364	5.64	–	–
Cyclin Dependent Kinase Inhibitor 1C	CDKN1C	ENSG00000129757	4.74	2.84	2.43
Decorin	DCN	ENSG00000011465	3.81	–	–
**UNCATEGORIZABLE GENES**					
Bone Morphogenetic Protein 6	BMP6	ENSG00000153162	5.49	2.18	-1.41
GULP, Engulfment Adaptor PTB Domain Containing 1	GULP1	ENSG00000144366	4.11	0.78	1.41
–	AC106865.1	ENSG00000250771	4.88	–	–
Contactin 3	CNTN3	ENSG00000113805	4.51	–	–
Ganglioside Induced Differentiation Associated Protein 1	GDAP1	ENSG00000104381	-3.93	-1.12	-0.63
Chromosome 1 Open Reading Frame 116	C1orf116	ENSG00000182795	-3.57	-1.47	1.23
Serine Dehydratase Like	SDSL	ENSG00000139410	-2.88	-0.68	-0.72

*Upregulated genes are represented in red, downregulated in blue, and those with no detectable changes with the symbol “-”. All genes curated had an FDR p-value < 4.0 × 10^−15^ except the following; A549's CDKN1C (0.01) and HEY1 (0.04), HCT116's BMP6 (0.05). Experiments were conducted in biological triplicates of n = 3. Confirmatory repeat experimental data can be found in Supplementary Table S1*.

The 21 DEGs revealed a “balancing act” in HSAEC between genes of oncogenic and tumor-suppressive nature. Oncogenic genes such as *PDK4, RRAGD, HEY1, TSPAN15*, and *SERPINB9* were found overexpressed. At the same time, tumor-suppressive genes such as *MT1G, MT1M, CDKN1C*, and *DCN* were overexpressed. On the other hand, genes of oncogenic nature such as *SHCBP1, CPA4, KRT19, KRT15, and DSG3* were found downregulated. DEGs such as *BMP6, GULP1, AC106865.1, CNTN3, GDAP1, C1orf116, and SDSL* were uncategorizable due to their lack of information.

### PG Possibly Induced DNA Replication Inhibition and Metabolic Rewiring in A549 and HCT116

To identify other possible anti-cancer mechanisms associated with PG, we performed a comparative transcriptomics analysis between A549, HCT116 and HSAEC cells treated with 1.0 μM PG for 24 h. A total of 18 DEGs were considered fit for comparison (Table 2) based on two conditions. Firstly, the DEGs commonly perturbed between A549 and HCT116 had to be upregulated by at least >2 Log2FC and downregulated by < -1.5 Log2FC. Secondly, the difference between A549 and HSAEC gene expression had to be >± 1.5 (Figure 2).

**Table 2:** Common DEGs in both A549 and HCT116 cells after 24 h treatment with 1.0 μM PG.

			**Log2FC**		
**Gene name**	**Gene symbol**	**ENSEMBL ID**	**HSAEC**	**A549**	**HCT116**
**DNA-REPLICATION ASSOCIATED GENES**					
Minichromosome Maintenance 10 Replication Initiation Factor	MCM10	ENSG00000065328	-4.67	-3.09	-1.87
H2A Histone Family Member X	H2AFX	ENSG00000188486	-0.96	-2.70	-1.57
DNA Replication and Sister Chromatid Cohesion 1	DSCC1	ENSG00000136982	-3.93	-2.35	-1.55
Minichromosome Maintenance Complex Component 4	MCM4	ENSG00000104738	-0.33	-2.22	-1.87
Replication Factor C Subunit 5	RFC5	ENSG00000111445	-0.51	-2.11	-1.62
**LIPID AND CHOLESTEROL METABOLISM ASSOCIATED GENES**					
Aldolase, Fructose-Bisphosphate C	ALDOC	ENSG00000109107	1.74	5.36	4.71
N-Myc Downstream Regulated 1	NDRG1	ENSG00000104419	1.08	3.80	2.81
WD Repeat Domain, Phosphoinositide Interacting 1	WIPI1	ENSG00000070540	1.53	3.39	2.50
Proprotein Convertase Subtilisin/Kexin Type 9	PCSK9	ENSG00000169174	1.65	3.27	2.85
Lipase G, Endothelial Type	LIPG	ENSG00000101670	-0.27	2.82	3.35
Methylsterol Monooxygenase 1	MSMO1	ENSG00000052802	0.51	2.76	3.32
Mevalonate Diphosphate Decarboxylase	MVD	ENSG00000167508	0.52	2.48	2.76
Isopentenyl-Diphosphate Delta Isomerase 1	IDI1	ENSG00000067064	0.59	2.34	2.98
Angiopoietin Like 4	ANGPTL4	ENSG00000167772	-1.34	2.19	3.66
**OTHER PATHWAYS ASSOCIATED GENES**					
MIR210 (MicroRNA 210) Host Gene	MIR210HG	ENSG00000247095	1.00	4.57	3.60
Cyclin G2	CCNG2	ENSG00000138764	0.78	3.15	3.74
Prolyl 4-Hydroxylase Subunit Alpha 1	P4HA1	ENSG00000122884	0.30	2.40	2.68
Protein Phosphatase, Mg2+/Mn2+ Dependent 1K	PPM1K	ENSG00000163644	0.57	2.32	2.15

*Upregulated genes are represented in red and downregulated in blue. All genes curated had an FDR p-value < 4.0 × 10^−15^ except the following; HSAEC's MIR210HG (4.09 × 10^−15^), LIPG (7.17 × 10^−3^), MSMO1 (2.15 × 10^−12^), MVD (5.92 × 10^−10^), P4HA1 (4.41 × 10^−4^), IDI1 (3.13 × 10^−10^), PPM1K (8.86 × 10^−3^), MCM10 (3.26 × 10^−7^), H2AFX (4.09 × 10^−15^), DSCC1 (8.96 × 10^−4^), MCM4 (2.54 × 10^−3^), and RCF5 (0.02). Experiments were conducted as biological triplicates of n = 3. Confirmatory repeat experimental data can be found in Supplementary Table S2*.

All commonly downregulated genes between A549 and HCT116 were found associated with DNA replication. These were *MCM10, H2AFX, DSCC1, MCM4*, and *RFC5* (Table 2). Surprisingly, *MCM10* and *DSCC1* expression were severely repressed in HSAEC than in A549 and HCT116. On the other hand, multiple genes associated with lipid and cholesterol metabolism, either directly or indirectly, were found commonly overexpressed between A549 and HCT116. These were *ALDOC, NDRG1, WIPI1, PCSK9, LIPG, MSMO1, MVD, IDI1*, and *ANGPTL4* (Table 2). The other genes that were overexpressed yet did not closely associate with the two main categories described here were *MIR210HG, CCNG2, P4HA1*, and *PPM1K* (Table 2). Confirmatory repeat experimental data for RNA sequencing result of A549 and HCT116 can be found in Tables S1,S2. Further pathway analysis also revealed different upstream regulator activities in PG-treated HSAEC, A549, and HCT116 cells (Tables S3–S5).

Based on pathway analysis, and in relation to DNA replication, the “Role of BRCA1 in DNA Damage Response” and the “Mitotic Roles of Polo-Like Kinase” pathways were seen perturbed in all three cell lines but were predicted to be inactivated (Table 3). In terms of DNA damage, the “Cell Cycle: G2/M DNA Damage Checkpoint Regulation” pathway was predicted to be activated (Table 3). In relation to metabolic rewiring, the “Superpathway of Cholesterol Biosynthesis,” the “Cholesterol Biosynthesis III (via Desmosterol),” the “Cholesterol Biosynthesis II (via 24,25-dihydrolanosterol),” and the “Cholesterol Biosynthesis I” pathways were significantly perturbed and predicted to be highly activated (Table 3). Furthermore, these cholesterol pathways were not perturbed in HSAEC following PG treatment.

**Table 3:** Top 10 canonical pathways in A549 and HCT116 cells after 24 h of 1.0 μM PG treatment.

**Top 10 Canonical pathways**	**-log(*****p-*****value)**			**Activation z-score**		
	**HSAEC**	**A549**	**HCT116**	**HSAEC**	**A549**	**HCT116**
	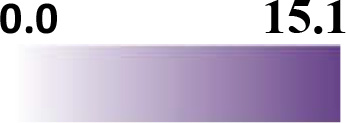			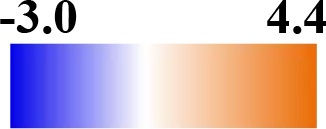		
Superpathway of Cholesterol Biosynthesis	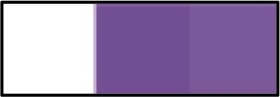			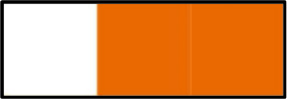		
Cell Cycle Control of Chromosomal Replication	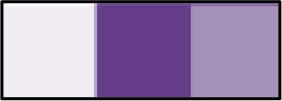			Not predictable		
Cholesterol Biosynthesis III (via Desmosterol)	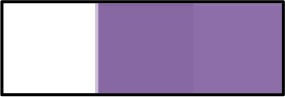			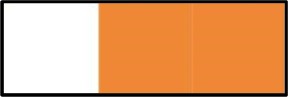		
Cholesterol Biosynthesis II (via 24,25-dihydrolanosterol)	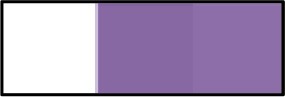			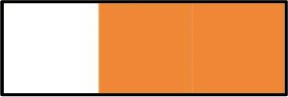		
Cholesterol Biosynthesis I	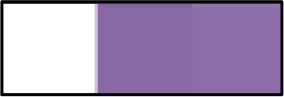			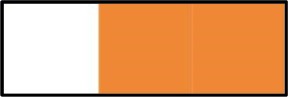		
Role of BRCA1 in DNA Damage Response	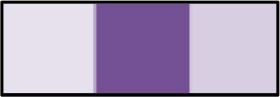			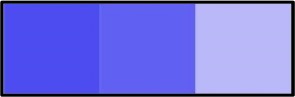		
Mitotic Roles of Polo-Like Kinase	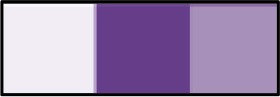			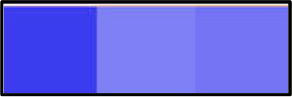		
Hereditary Breast Cancer Signaling	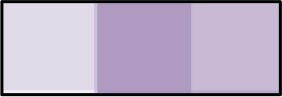			Not predictable		
Mismatch Repair in Eukaryotes	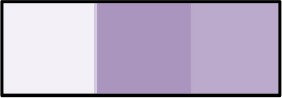			Not predictable		
Cell Cycle: G2/M DNA Damage Checkpoint Regulation	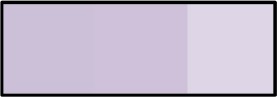			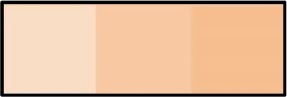		

*Pathways were ranked in descending order of decreasing -log(p-value) of the Fisher's exact test. Dark purple heat-map blocks represent high -log(p-value). Activation z-scores were calculated based on the IPA's pathway activity prediction algorithm. Dark orange heat-map blocks represent the possibility of a highly active pathway, whereas dark blue blocks represent inhibition*.

**Figure 3:**
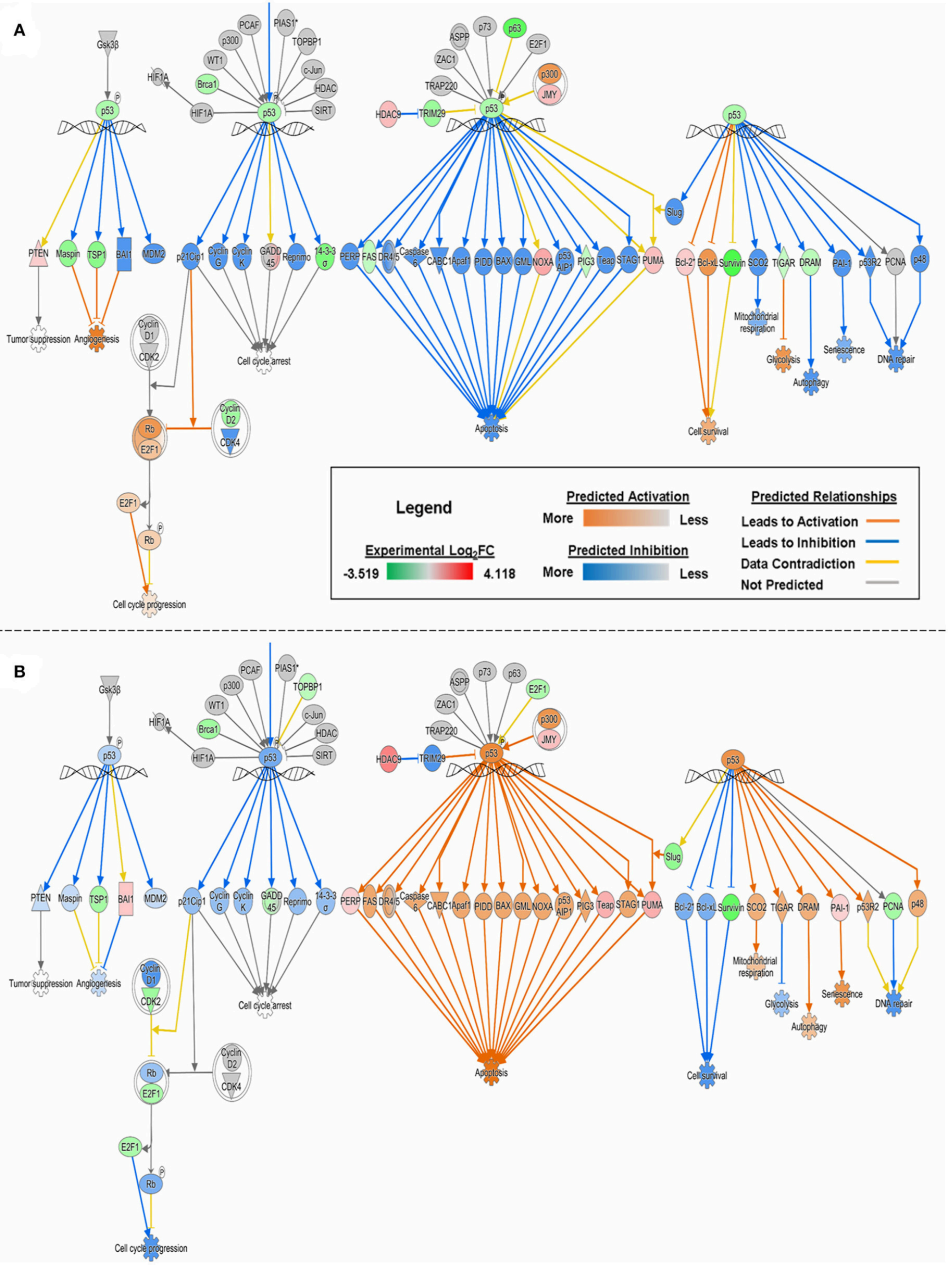
MAP of “p53 Signaling” pathway in **(A)** HSAEC and **(B)** A549 cells treated with 1 μM PG over 24 h. Predictions were calculated based upon DEGs from the experimental dataset overlaid onto the Ingenuity Knowledge Base in IPA. Orange, blue, yellow, and gray lines corresponds to predicted activation, inhibition, contradiction, and the inability to predict an outcome, respectively. Red or green color intensities within shapes reflect the level of upregulation or downregulation, respectively, based upon the experimental Log2FC values. Orange or blue color intensities within shapes reflect the level of predicted activation or inhibition, respectively, based on upon IPA's predictions.

With experimental data, the IPA's Molecule Activity Prediction (MAP) algorithm managed to predict PG-induced mechanistic differences between HSAEC and A549 cells in terms of “Cell Cycle Progression,” “Apoptosis,” “Cell Survival,” “Mitochondrial Respiration,” “Glycolysis,” “Autophagy,” and “Senescence” (Figure 3). The overall prediction landscape seems to suggest PG-induced pro-survival in HSAEC but pro-death in A549. Interestingly, “DNA Repair” mechanism was predicted to be inhibited in both cell lines (Figure 3).

## Discussion

Metastatic lung cancers are extremely lethal and requires effective systemic therapies to improve clinical outcomes for patients (46). PG has demonstrated immense potential as a smart chemotherapeutic candidate. Its most promising feature is its ability to selectively eliminate cancer cells yet protect healthy cells (37–39). Here, we were able to demonstrate PG's selective elimination of NSCLC by four-folds (Figures 1A–C). Beyond lung adenocarcinoma cells, we also showed that PG could cause substantial morphological alterations to colorectal carcinoma cells (Figure 1D). When compared to other naturally derived anti-cancer agents such as DTX, PTX, or DOX, PG exhibited heightened protection toward HSAEC while being more toxic to A549. Indeed, PG established itself as a promising cancer-specific agent. However, the random combination with other anti-cancer agents could ameliorate PG's cancer-specific activity and yield an undesirable outcome to healthy cells (Figures 1A–C). A rational drug combination approach could increase synergism, hence, greater success in combinatorial chemotherapies. To permit a rational combination of PG with other anti-cancer agents, we require a deeper understanding of the agent's molecular functions.

Previously, a microarray analysis for 1,176 genes was performed on human breast cancer cells treated with PG (44). Out of the 37 significantly perturbed genes (44), there were no similarities found with our study (Table 2). The lack of similarities was not unexpected as this could be due to the inherent limitation of the microarray technology (47), or simply because a different cell line was used. Nevertheless, using RNA-sequencing, a genome-wide transcriptomics approach, we were able to identify at least 2,000 significantly perturbed genes per cell line. With broader coverage, we were confident that employing such a technology would permit a more comprehensive analysis.

The comparative transcriptomics analysis between A549 and HCT116 revealed 18 genes that were significantly perturbed by PG (Table 2). These genes revealed the possibility of DNA replication inhibition and metabolic rewiring toward enhanced lipid and cholesterol biogenesis. In the study with breast cancer cells, PG was reported to perturb genes related to transcriptional regulation, cell adhesion, cell cycle, and apoptosis (44). Although we have not found perturbations in genes associated with transcriptional regulation or cell adhesion, based on experimental data, we have predicted cell cycle inhibition (Table 3 and Figure 3) and reduced survival fitness in line with apoptosis (Figure 3) in A549 cells.

The gene products of *MCM10, MCM4, H2AFX, DSCC1*, and *RFC5* are necessary for DNA replication. However, they were found downregulated in both A549 and HCT116 after PG treatment (Table 2). MCM10 plays a crucial role in allowing CDC45:MCM2-7:GINS helicase to unwind DNA double-strand for replication initiation (48). After DNA has been unwounded, DNA replication requires DSCC1 and RFC5 complexed with other proteins to load Proliferating Cell Nuclear Antigen (PCNA) onto the DNA (49). PCNA is required to clamp DNA polymerase epsilon onto the DNA for replication (50). After DNA synthesis, to maintain genomic integrity, H2AFX serves as a sensor for DNA damage and recruits DNA repair complexes to the area of lesion (51). PG has been reported to cause genotoxicity directly through copper-mediated oxidative cleavage (52), or indirectly through inhibition of topoisomerases (53). One potential mechanism stemming from the downregulation of *H2AFX* is the loss of genomic integrity, induction of cell cycle arrest [*CCNG2* overexpression (Table 2) and predicted G2/M DNA damage checkpoint arrest activation (Table 3)] and therefore, DNA replication stand-still (54, 55). By throwing the DNA repair mechanisms off-balance [predicted BRCA pathway shutdown (Table 3)], genotoxic agents such as PG might increase sensitivity and effectiveness against cancer cells (56, 57).

Metabolic rewiring has been described as an emerging hallmark of cancer (58, 59), and there have been reports of lipid and cholesterol metabolism being drivers of tumorigenesis and progression (60–62). In fact, it has been mentioned that “highly proliferative cancer cells show a strong lipid and cholesterol avidity, which they satisfy by either increasing the uptake of exogenous (or dietary) lipids and lipoproteins or overactivating their endogenous synthesis (that is, lipogenesis and cholesterol synthesis, respectively)” (60). Interestingly, these overactivations were observed only after PG treatment (Table 2). ALDOC, MVD, and IDI1 are metabolic enzymes that support lipid and cholesterol biosynthesis. Their gene overexpression could potentially hint at an overactive endogenous lipid and cholesterol biogenesis. *ANGPTL4*, a lipoprotein lipase inhibitor, had a Log2FC difference of 3.53 between healthy HSAEC and cancerous A549 cells. *ANGPTL4* upregulation in A549 cells may have been in response to the overexpression of other lipogenic genes (63). On the flip side, upregulation of *PCKS9* hints at a potential supply cut-off of low-density lipoproteins (LDL) from exogenous sources by reducing LDL receptors (64, 65). As a compensatory mechanism to reduced LDL uptake, *NDRG1* and *LIPG* may have been upregulated to acquire LDL and fatty acids, respectively, from the cell's surroundings (66, 67). *CXCL8*, otherwise known as interleukin-8, has been implicated as a cancer growth factor (68, 69), as well as a molecule that promotes cholesterol accumulation (70). *MSMO1* is also believed to be involved in cholesterol metabolism and cancer (71, 72). Altogether, there may be a possibility that the blockade of exogenous LDL import, compounded with the rampant endogenous demand for lipid and cholesterol biogenesis to support rapidly dividing cancer cells, induced a suicidal metabolic rewiring that eventually led to autophagy (73).

Autophagy is a form of cellular self-cannibalization of cytoplasmic content via lysosomal compartments to recycle cell materials and provide substrates for cellular homeostasis under metabolic stress (74). However, autophagy can be a double-edged sword when it comes to cancers. It could either be pro- tumorigenic or anti-tumorigenic (75, 76). PG is known to bind and inhibit mTORC1 and mTORC2, initiating autophagy in cancer cells (39, 77, 78). We found *WIPI1*, a marker and an important player in autophagy (79, 80), markedly upregulated (Table 2). It is unclear if the lipid and cholesterol biosynthesis genes were upregulated to support the *de novo* biogenesis of autophagosomes.

How PG protects healthy cells yet eliminates cancer cells has been a mystery thus far. For the first time, we attempted to unravel PG's cancer-specific mechanisms of action through comparative transcriptomics analysis. Firstly, unlike in A549 and HCT116, there were little to no upregulation in lipid and cholesterol biosynthetic genes and pathways in HSAEC (Tables 2, 3). In fact, the downregulation of *ANGPTL4* suggests an active catabolism of lipoproteins. Secondly, although *WIPI1* was upregulated, it was much lesser than A549, possibly reflecting a weaker autophagic status in HSAEC. Thirdly, the near-normal expression of *H2AFX* suggests that HSAEC may be able to overcome PG's genotoxic stress. However, how this could be possible despite *BRCA1* downregulation (Figure 3) and potential BRCA1 pathway inactivation (Table 3) is unclear. Fourth, a deep analysis of HSAEC-specific genes perturbed by PG revealed a “balancing act” expression of pro-cancer and anti-cancer genes (Table 1). This could potentially assist in HSAEC's viability under PG treatment. Lastly, and surprisingly, *MCM10* and *DSCC1* were found severely downregulated in HSAEC. As PG could inhibit topoisomerases (53), another potential means of PG genotoxicity could be mitigated here as the loss of MCM10 does not permit DNA to unwind for replication (48). Altogether, we suspect that HSAEC may have been conferred protection to PG through DNA replication inhibition, BRCA1-independent DNA repair availability and autophagic resistance.

PG's upregulation of cholesterol pathways in cancer cells and its ability to potentially inhibit DNA replication brings about two immediate concerns that should be addressed in future studies. Firstly, the degree of which PG could inhibit DNA replication in HSAEC should be monitored with cell growth rate compared to A549 and other rapidly dividing cells. This would elucidate the potential clinical benefits PG has over other conventional chemotherapeutics that falls short in protecting rapidly dividing healthy cells. Secondly, the impact of PG treatment with regards to hypercholesterolemia should be assessed *in vivo*. On the other hand, further studies on *MIR210HG*, the second most differentially expressed gene in both A549 and HCT116 (Table 2) could potentially highlight novel insights with regards to PG's cancer-specific mechanisms of action. To further improve PG's cancer specificity, chemical modifications may be explored to acquire novel PG analogs or develop targeted drug delivery strategies which studies have already begun (81, 82).

## Conclusion

Numerous decades of cancer research, drug discovery, and development have led to major improvements in patients' quality of life. Research into systemic therapies for metastatic cancers continues at two major fronts, namely, safety and efficacy. PG appears to be a promising smart chemotherapeutic agent against NSCLC. PG not only demonstrated heightened anti-cancer activity against A549, but this activity was also cancer-specific. Understanding how such an agent differentiates cancerous from healthy cells has been unclear until recently. With RNA-sequencing, a next-generation tool for transcriptomics, we managed to unravel PG's potential cancer-specific mechanisms of action. Through an exogenous cholesterol supply cut-off and an internal overactivation of cholesterol synthesis, PG might have induced cancer cell autophagy to a point whereby self-cannibalization led to cell death. At the same time, through balancing the overexpression of oncogenic and tumor-suppressive genes, healthy cells might have been conferred a heightened survival status by PG. By exposing A549 transcriptome landscape perturbed by PG, we can now conduct further experiments with single or multiplexed knock-outs and knock-downs using CRISPR to yield definitive targets which could aid the development of precision medicine against NSCLC.

## Author Contributions

BD performed the cell cultures, cytotoxicity assays, RNA extraction, RNA purification, RNA-sequencing data processing in CLC Workbench, and the data analysis in IPA. JN repeated the RNA extraction throughout data analysis in IPA and reproduced the data. LL, QX, and LY conceptualized the study idea and provided material and technical support. BD and LL wrote the manuscript. BD, JN, QX, LL, and LY revised the manuscript.

### Conflict of Interest Statement

The authors declare that the research was conducted in the absence of any commercial or financial relationships that could be construed as a potential conflict of interest.
