# Supplementary material for: Comparative Transcriptomics Unravels Prodigiosin's Potential Cancer-Specific Activity Between Human Small Airway Epithelial Cells and Lung Adenocarcinoma Cells
Source: Front Oncol. 2018 Dec 5;8:573. doi: 10.3389/fonc.2018.00573 (PMC6290060; doi:10.3389/fonc.2018.00573)
Supplement: Supplementary file 1 [file Data_Sheet_1.docx]

Supplementary Material

**Comparative Transcriptomics Unravels Prodigiosin’s Potential Cancer-Specific Activity Between Human Small Airway Epithelial Cells and Lung Adenocarcinoma Cells**

**Bala Davient^1, 2^, Jessica Pei Zhen Ng^1, 2^, *Qiang Xiao^3^, *Liang Li^4, 2^, and *Liang Yang^1, 2, 5^**

**^1^Singapore Centre for Environmental Life Sciences Engineering, Nanyang Technological University, 60 Nanyang Dr, Singapore, 637551.**

**^2^School of Biological Sciences, Nanyang Technological University, 60 Nanyang Dr, Singapore, 637551.**

**^3^Respiratory medicine, Shunde Hospital, Southern Medical University (The First People's Hospital of Shunde Foshan), No.1 Jiazi Road, Lunjiao, Shunde District, Foshan City, Guangdong Province, China, 528308.**

**^4^Shenzhen Institute of Advance Technology, Chinese Academy of Sciences, Shenzhen, China, 518055.**

**^5^School of Medicine, Southern University of Science and Technology, Shenzhen, China, 518055.**

*** Correspondence:**Dr. Liang Li Dr. Liang Yang Dr. Qiang Xiao
[liang.li@siat.ac.cn](mailto:liang.li@siat.ac.cn) [yangl@sustc.edu.cn](mailto:yangl@sustc.edu.cn) [xiaoq@mail2.sysu.edu.cn](mailto:xiaoq@mail2.sysu.edu.cn)

**
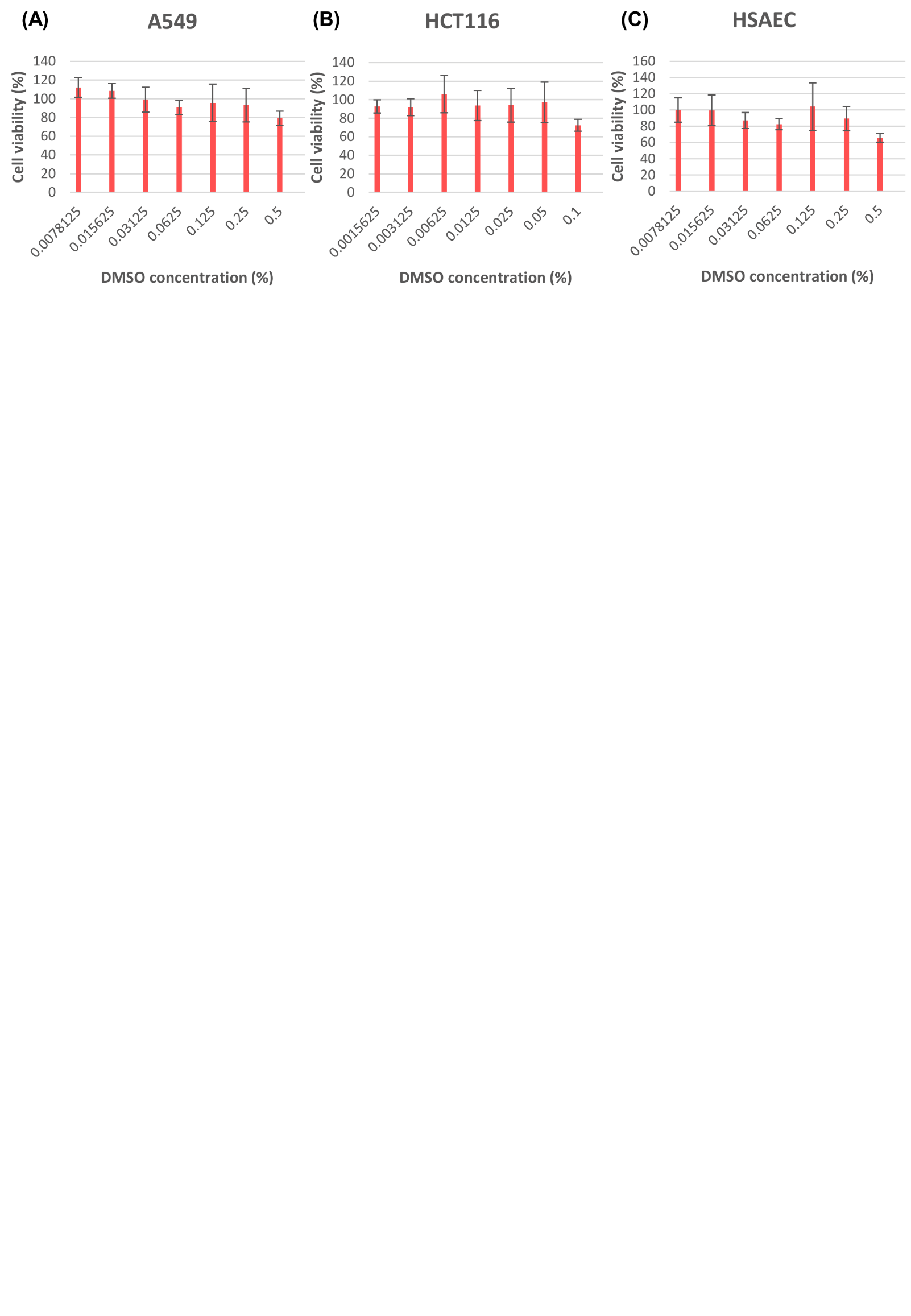
**

**Figure S1. Cell viability of (A) A549, (B) HCT116 and (C) HSAEC measured by the MTT assay after 48-hours of various DMSO concentrations.** Bar graphs represent mean cell viability from biological triplicates while the black lines on the bar tops represent standard deviation (SD). Experiments were in biological triplicates of *n* = 3. Note: 1.0µM PG contains 0.05% DMSO.


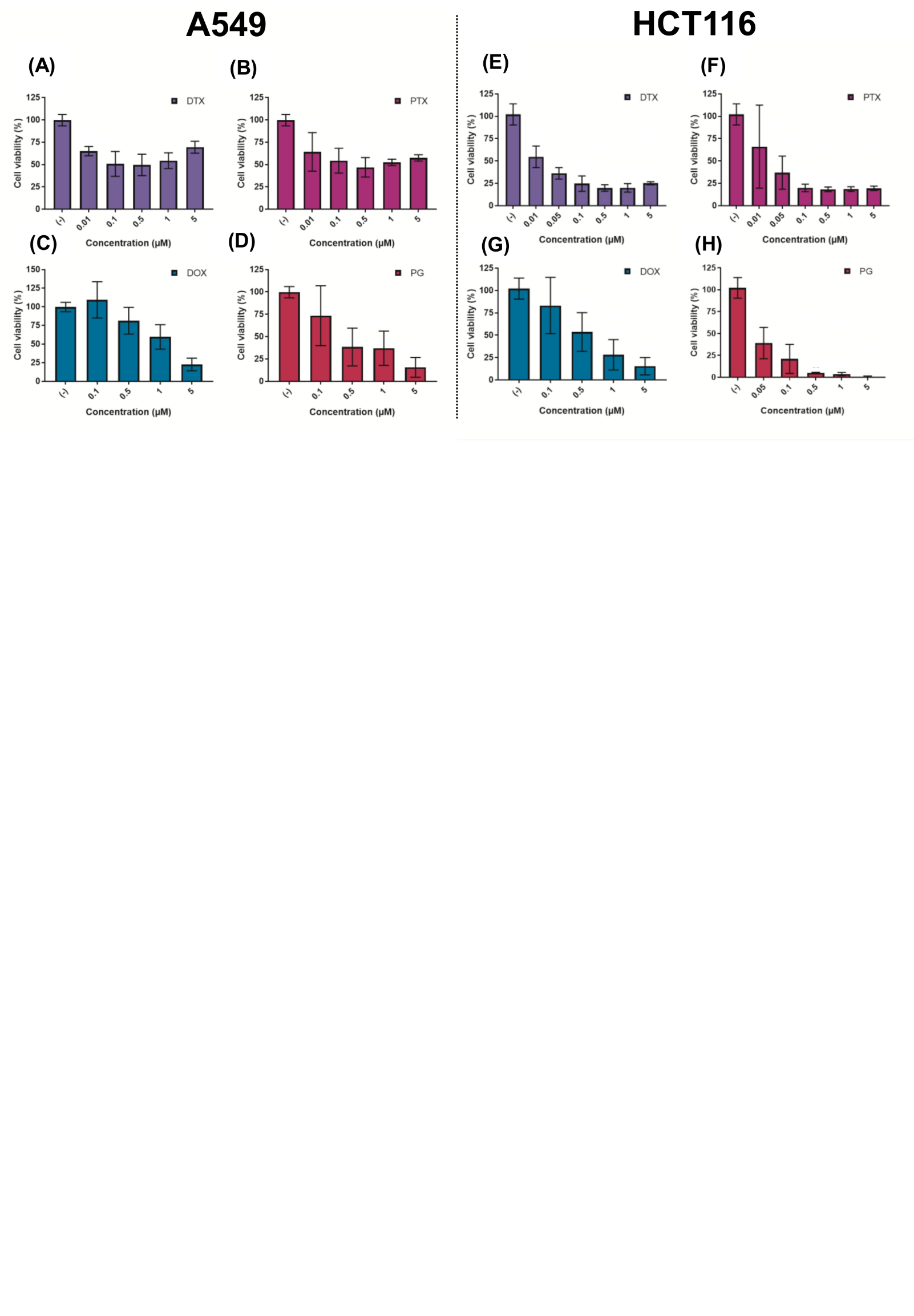
**Figure S2. Effects of DTX, PTX, DOX and PG on A549 and HCT116 cell viability in comparison to 0.3% DMSO negative (-) control 48 hours post-treatment.** (A) DTX vs A549, (B) PTX vs A549, (C) DOX vs A549, (D) PG vs A549, (E) DTX vs HCT116, (F) PTX vs HCT116, (G) DOX vs HCT116, (H) PG vs HCT116. Drug challenge were between the range of 0.01µM to 5µM. Data represent mean cell viability ± standard deviation (SD). Experiments were carried out in biological triplicates of *n* = 3.

**Table S1. Differentially expressed genes of A549 and HCT116 at Passage 10 after 24 hours treatment with 1.0µM PG (for gene entries in Table 1, the HSAEC list) compared against a repeated experiment with A549 and HCT116 at Passage 24 (from a different cryovial).** Upregulated genes are represented in red, downregulated in blue, and those with no detectable changes in grey. HSAEC cell line was unavailable at the point in time for ‘Batch 2’ repeat experiment. Not all genes in comparison between both batches achieved an FDR p-value < 4.0x10^-15^. Batch 1 (n=3) and Batch 2 (n=1) RNA extraction and downstream processing were independently performed by two researchers utilizing an identical protocol.

| **Gene Symbol** | **ENSEMBL ID** | **Log2 FC** | | | |
| --- | --- | --- | --- | --- | --- |
|  |  | **Batch 1  (as in Table 1)** | | **Batch 2**  **(Repeated, *n=*1)** | |
|  |  | **A549** | **HCT116** | **A549** | **HCT116** |
| PDK4 | ENSG00000004799 | 1.25 | 1.62 | 1.27 | - |
| MT1G | ENSG00000125144 | - | - | **-** | - |
| MT1M | ENSG00000205364 | - | - | **-** | - |
| BMP6 | ENSG00000153162 | 2.18 | -1.41 | 2.21 | 0.88 |
| RRAGD | ENSG00000025039 | 2.03 | 0.68 | 2.66 | - |
| AC106865.1 | ENSG00000250771 | - | - | **-** | - |
| CDKN1C | ENSG00000129757 | 2.84 | 2.43 | **-** | - |
| HEY1 | ENSG00000164683 | 0.93 | 1.41 | 2.98 | - |
| CNTN3 | ENSG00000113805 | - | - | 0.97 | - |
| TSPAN15 | ENSG00000099282 | -0.61 | 1.28 | **-** | - |
| GULP1 | ENSG00000144366 | 0.78 | 1.41 | -0.69 | - |
| DCN | ENSG00000011465 | - | - | 1.25 | - |
| SERPINB9 | ENSG00000170542 | -0.78 | 0.72 | **-** | - |
| GDAP1 | ENSG00000104381 | -1.12 | -0.63 | -0.65 | - |
| SHCBP1 | ENSG00000171241 | -2.18 | -1.14 | -1.25 | 1.32 |
| CPA4 | ENSG00000128510 | 0.49 | 0.67 | -2.71 | 0.89 |
| C1orf116 | ENSG00000182795 | -1.47 | 1.23 | -2.98 | 3.14 |
| KRT19 | ENSG00000171345 | 1.97 | 0.76 | -1.48 | - |
| KRT15 | ENSG00000171346 | 1.13 | 2.00 | 1.79 | - |
| SDSL | ENSG00000139410 | -0.68 | -0.72 | 1.13 | - |
| DSG3 | ENSG00000134757 | - | - | -0.59 | 0.58 |

**Table S2. Differentially expressed genes of A549 and HCT116 at Passage 10 after 24 hours treatment with 1.0µM PG (for gene entries in Table 2, the cancer cell list) compared against a repeated experiment with A549 and HCT116 at Passage 24 (from a different cryovial).** Upregulated genes are represented in red and downregulated in blue. All genes curated had an FDR p-value < 4.0x10^-15^. Batch 1 (n=3) and Batch 2 (n=1) RNA extraction and downstream processing were independently performed by two researchers utilizing an identical protocol.

| **Gene Symbol** | **ENSEMBL ID** | **Log2 FC** | | | |
| --- | --- | --- | --- | --- | --- |
|  |  | **Batch 1  (as in Table 2)** | | **Batch 2**  **(Repeated, *n=*1)** | |
|  |  | **A549** | **HCT116** | **A549** | **HCT116** |
| ALDOC | ENSG00000109107 | 5.36 | 4.71 | 6.35 | 4.70 |
| MIR210HG | ENSG00000247095 | 4.57 | 3.60 | 4.75 | 3.69 |
| NDRG1 | ENSG00000104419 | 3.80 | 2.81 | 4.01 | 3.13 |
| WIPI1 | ENSG00000070540 | 3.39 | 2.50 | 4.44 | 2.84 |
| PCSK9 | ENSG00000169174 | 3.27 | 2.85 | 3.24 | 3.16 |
| CCNG2 | ENSG00000138764 | 3.15 | 3.74 | 3.54 | 3.37 |
| LIPG | ENSG00000101670 | 2.82 | 3.35 | 2.94 | 3.93 |
| MSMO1 | ENSG00000052802 | 2.76 | 3.32 | 3.29 | 3.33 |
| MVD | ENSG00000167508 | 2.48 | 2.76 | 3.23 | 2.75 |
| P4HA1 | ENSG00000122884 | 2.40 | 2.68 | 2.60 | 2.39 |
| IDI1 | ENSG00000067064 | 2.34 | 2.98 | 2.80 | 2.93 |
| PPM1K | ENSG00000163644 | 2.32 | 2.15 | 3.03 | 2.16 |
| ANGPTL4 | ENSG00000167772 | 2.19 | 3.66 | 2.12 | 4.21 |
| MCM10 | ENSG00000065328 | -3.09 | -1.87 | -4.19 | -2.43 |
| H2AFX | ENSG00000188486 | -2.70 | -1.57 | -2.91 | -1.53 |
| DSCC1 | ENSG00000136982 | -2.35 | -1.55 | -2.99 | -2.37 |
| MCM4 | ENSG00000104738 | -2.22 | -1.87 | -2.99 | -2.49 |
| RFC5 | ENSG00000111445 | -2.11 | -1.62 | -2.51 | -1.96 |

**Table S3. Top 20 IPA-predicted upstream regulators and activity of HSAEC after 24 hours treatment with 1.0µM PG.**

| **HSAEC** | | | | | |
| --- | --- | --- | --- | --- | --- |
| **Upstream Regulator** | **Molecule Type** | **Log2 FC** | ***p-*value of overlap** | **Activation z-score** | **Predicted Activation State** |
| beta-estradiol | chemical - endogenous mammalian | - | 6.37E-51 | -0.67 | - |
| TNF | cytokine | 1.45 | 7.74E-44 | 3.49 | Activated |
| TP53 | transcription regulator | -1.44 | 7.85E-42 | 1.45 | - |
| TGFB1 | growth factor | 0.74 | 1.76E-34 | 1.26 | - |
| Vegf | group | - | 5.52E-34 | -1.10 | - |
| HGF | growth factor | 3.26 | 1.98E-33 | -1.19 | - |
| dexamethasone | chemical drug | - | 2.83E-33 | 2.91 | Activated |
| ERBB2 | kinase | -0.95 | 3.2E-33 | -2.50 | Inhibited |
| calcitriol | chemical drug | - | 8.67E-33 | 4.02 | Activated |
| IL1B | cytokine | 0.47 | 2.1E-32 | 4.48 | Activated |
| lipopolysaccharide | chemical drug | - | 1.19E-30 | 2.09 | Activated |
| IFNG | cytokine | - | 4.04E-30 | 1.65 | - |
| NUPR1 | transcription regulator | 1.65 | 1.74E-29 | 7.45 | Activated |
| ESR1 | ligand-dependent nuclear receptor | -3.70 | 1.07E-28 | -3.35 | Inhibited |
| CSF2 | cytokine | 0.33 | 6.02E-28 | -2.04 | Inhibited |
| PD98059 | chemical - kinase inhibitor | - | 7.27E-27 | -2.85 | Inhibited |
| tretinoin | chemical - endogenous mammalian | - | 3.54E-25 | 3.13 | Activated |
| FOXO3 | transcription regulator | 0.46 | 4.33E-25 | 1.15 | - |
| progesterone | chemical - endogenous mammalian | - | 4.58E-25 | 1.33 | - |
| dextran sulfate | chemical drug | - | 1.61E-24 | 0.67 | - |

**Table S4. Top 20 IPA-predicted upstream regulators and activity of A549 after 24 hours treatment with 1.0µM PG.**

| **A549** | | | | | |
| --- | --- | --- | --- | --- | --- |
| **Upstream Regulator** | **Molecule Type** | **Log2 FC** | ***p-*value of overlap** | **Activation z-score** | **Predicted Activation State** |
| TP53 | transcription regulator | -0.44 | 4.42E-50 | 6.34 | Activated |
| E2F4 | transcription regulator | -0.53 | 1.24E-46 | -0.59 | - |
| ERBB2 | kinase | 0.06 | 2.13E-45 | -3.83 | Inhibited |
| CDKN1A | kinase | 0.27 | 1.29E-44 | 3.52 | Activated |
| NUPR1 | transcription regulator | 2.07 | 8.92E-41 | 9.50 | Activated |
| HGF | growth factor | -3.09 | 4.43E-38 | -0.23 | - |
| E2F1 | transcription regulator | -1.43 | 3.96E-37 | -4.16 | Inhibited |
| Vegf | group | - | 8.34E-37 | -0.57 | - |
| dextran sulfate | chemical drug | - | 1.84E-35 | 0.44 | - |
| CSF2 | cytokine | 0.11 | 4.89E-35 | -4.11 | Inhibited |
| CCND1 | transcription regulator | -0.18 | 6.1E-35 | -3.87 | Inhibited |
| TBX2 | transcription regulator | 0.15 | 3.96E-33 | -6.14 | Inhibited |
| discodermolide | chemical drug | - | 6.31E-33 |  | - |
| FOXO3 | transcription regulator | 1.29 | 1.82E-31 | 1.72 | - |
| RABL6 | other | -0.70 | 2.94E-31 | -6.33 | Inhibited |
| beta-estradiol | chemical - endogenous mammalian | - | 4.74E-31 | -0.31 | - |
| methylselenic acid | chemical reagent | - | 2.22E-29 |  | - |
| calcitriol | chemical drug | - | 1.79E-28 | 5.59 | Activated |
| ESR1 | ligand-dependent nuclear receptor | -0.77 | 4.31E-28 | -6.53 | Inhibited |
| fulvestrant | chemical drug | - | 3.45E-26 | 4.29 | Activated |

**Table S5. Top 20 IPA-predicted upstream regulators and activity of HCT116 after 24 hours treatment with 1.0µM PG.**

| **HCT116** | | | | | |
| --- | --- | --- | --- | --- | --- |
| **Upstream Regulator** | **Molecule Type** | **Log2 FC** | ***p-*value of overlap** | **Activation z-score** | **Predicted Activation State** |
| TP53 | transcription regulator | 0.36 | 4.55E-31 | 6.98 | Activated |
| NUPR1 | transcription regulator | 0.82 | 7.05E-31 | 7.74 | Activated |
| ERBB2 | kinase | -0.11 | 7.82E-29 | -2.70 | Inhibited |
| beta-estradiol | chemical - endogenous mammalian | - | 2.11E-23 | -1.09 | - |
| FOXO3 | transcription regulator | 1.42 | 1.29E-22 | 3.06 | Activated |
| ESR1 | ligand-dependent nuclear receptor | -0.07 | 7.03E-22 | -4.28 | Inhibited |
| E2F4 | transcription regulator | -0.74 | 7.62E-22 | -0.35 | - |
| Vegf | group | - | 9.44E-22 | 0.96 | - |
| E2F1 | transcription regulator | -0.67 | 1.94E-21 | -1.72 | - |
| SCAP | other | -0.31 | 1.53E-20 | 5.56 | Activated |
| RABL6 | other | -0.62 | 3.71E-20 | -5.67 | Inhibited |
| HGF | growth factor | - | 2.54E-19 | 0.41 | - |
| chloropromazine | chemical drug | - | 4.85E-19 | 1.72 | - |
| SREBF2 | transcription regulator | 1.62 | 1.13E-18 | 4.97 | Activated |
| calcitriol | chemical drug | - | 4.05E-18 | 5.09 | Activated |
| let-7 | microrna | - | 1.09E-17 | 5.44 | Activated |
| CCND1 | transcription regulator | -0.96 | 1.44E-16 | -1.67 | - |
| ADRB | group | - | 1.56E-16 | -3.27 | Inhibited |
| CDKN1A | kinase | 2.24 | 2.07E-16 | 2.09 | Activated |
| CSF2 | cytokine | 3.21 | 2.28E-16 | -2.59 | Inhibited |
